# Adipose Tissue Inflammation Induces B Cell Inflammation and Decreases B Cell Function in Aging

**DOI:** 10.3389/fimmu.2017.01003

**Published:** 2017-08-28

**Authors:** Daniela Frasca, Bonnie B. Blomberg

**Affiliations:** ^1^Department of Microbiology and Immunology, University of Miami Miller School of Medicine, Miami, FL, United States

**Keywords:** aging, obesity, inflammation, immunity, antibody responses

## Abstract

Aging is the greatest risk factor for developing chronic diseases. Inflamm-aging, the age-related increase in low-grade chronic inflammation, may be a common link in age-related diseases. This review summarizes recent published data on potential cellular and molecular mechanisms of the age-related increase in inflammation, and how these contribute to decreased humoral immune responses in aged mice and humans. Briefly, we cover how aging and related inflammation decrease antibody responses in mice and humans, and how obesity contributes to the mechanisms for aging through increased inflammation. We also report data in the literature showing adipose tissue infiltration with immune cells and how these cells are recruited and contribute to local and systemic inflammation. We show that several types of immune cells infiltrate the adipose tissue and these include macrophages, neutrophils, NK cells, innate lymphoid cells, eosinophils, T cells, B1, and B2 cells. Our main focus is how the adipose tissue affects immune responses, in particular B cell responses and antibody production. The role of leptin in generating inflammation and decreased B cell responses is also discussed. We report data published by us and by other groups showing that the adipose tissue generates pro-inflammatory B cell subsets which induce pro-inflammatory T cells, promote insulin resistance, and secrete pathogenic autoimmune antibodies.

## Aging and Related Inflammation Decrease Antibody Responses in Mice and Humans

Aged mice and humans have a poor immune response against infectious agents and vaccines (1). The antibody-mediated humoral immune response is qualitatively deficient with the production of antibodies of lower affinity (2–5) and with self-reactivity (6–8). Defects in T cells (9–11), B cells (12, 13), and antigen-presenting cells (14, 15) have been reported and all contribute to the age-related decrease in antibody production.

Our laboratory has characterized age-related autonomous B cell defects, which are responsible for sub-optimal antibody responses of elderly individuals to infections and vaccines (16–20). These include a reduction in activation-induced cytidine deaminase (AID), the enzyme necessary for class switch recombination, somatic hypermutation, and IgG production, as well as in E47 (13, 21), a key transcription factor regulating AID (22). Because AID correlates with optimal B cell function, it can be used as a predictive marker of optimal B cell response in humans (16, 17). The decrease in AID (4) leads to a reduced ability to generate higher affinity antibodies, e.g., to the influenza vaccine.

Aging is characterized by “inflamm-aging” (23), a low-grade chronic inflammation, which is a risk factor for morbidity and mortality of elderly individuals as it is implicated in the pathogenesis of several disabling diseases of the elderly such as type-2 diabetes mellitus (24), osteoporosis (25), Alzheimer’s disease (26), rheumatoid arthritis (27), and coronary heart disease (28). Our results have shown B cell functional deficiencies with increased inflammation with age in both mice (29) and humans (19). In particular, we demonstrated that increased TNF-α either in serum or in B cells contributes to sub-optimal antibody responses and we consider this to be a condition where the B cells have been made “refractory” to further stimulation by chronic stimulation with inflammatory cytokines.

## Obesity as a Mechanism of Aging

Among risk factors associated with disability and frailty, obesity seems to be a major contributor. Obesity is an inflammatory condition in which the innate immune system is chronically activated. Obesity contributes to pathologic conditions such as type-2 diabetes mellitus (30–32), cancer (33), psoriasis (34), atherosclerosis (35), and inflammatory bowel disease (36). Obesity is associated with sub-optimal immune responses in mice (37, 38) and humans (39).

The adipose tissue is generally separated into visceral adipose tissue (VAT) and subcutaneous adipose tissue (SAT) (40). The SAT accounts for ~80% of human adipose tissue, but the VAT is more metabolically active (41), and VAT accumulation is a greater predictor of obesity-associated mortality.

Fat mass increases with age in mice (42–44) and humans (45) and this is associated with low-grade chronic inflammation which contributes to the development of insulin resistance (IR) which also increases with age. Aging induces a significant increase in fat mass, redistribution of body fat with increased VAT, and decreased SAT, as well as ectopic VAT deposition. All these are strongly associated with worse health conditions in healthy elderly individuals (46). Moreover, aging may significantly affect AT function by changing the profile of inflammatory mediators produced by the adipocytes, modifying pre-adipocyte number and function and AT infiltration of macrophages (46), and other lymphocytes (see below).

It has recently been proposed that increased cellular stress in the adipocytes with age induces cellular senescence, which in turn leads to impaired removal of lipotoxic fatty acids, and increased secretion of pro-inflammatory cytokines and chemokines, due to the activation of the innate, and adaptive immune systems (47). These pro-inflammatory processes not only amplify each other but also have systemic consequences. These results suggest that cellular senescence is a stress-induced adaptive response that develops through major metabolic and secretory readjustments. This can occur at any time during life.

Studies in humans have shown that individuals with higher total and abdominal adiposity have shorter telomeres (48), suggesting that obesity may accelerate the aging process. Telomere length is inversely associated with body mass index (BMI), waist to hip ratio, independently of sex, age, fasting glucose and insulin, lipid and lipoprotein concentrations, physical activity, smoking status, and other metabolic risk factors.

## Adipose Tissue Inflammation

The AT is a major immunologically active organ that contributes to systemic inflammation through the secretion of pro-inflammatory cytokines and chemokines, as well as adipokines (49). Immune cells represent two-thirds of the stromal vascular fraction, and therefore the expansion of the AT during high-fat diet increases its ability to act as an immunological organ able to control systemic inflammation and metabolism. Chronic inflammation and immune cell infiltration in the AT are hallmark of obesity-associated IR and glucose intolerance.

Increased inflammation in the AT is the result of increased intrinsic inflammation in the adipocytes, which operates in a positive feedback loop, whereby the accumulation of infiltrating immune cells secrete pro-inflammatory cytokines and chemokines following interaction with the adipocytes. This feedback loop explains not only local but also systemic inflammation *via* the circulating immune cells. Infiltrating immune cells are drawn to the AT and become more inflammatory and these cells would generate sub-optimal immune responses in obesity by circulating to the peripheral lymphoid organs.

Immune cells infiltrating the AT include macrophages, neutrophils, NK cells, innate lymphoid cells (ILCs), eosinophils, T cells, B1, and B2 cells. The cellular composition of AT is dynamic and is regulated by acute and chronic stimuli including diet, body weight, fasting. In general, neutrophils are the first cells that infiltrate the expanding AT during high-fat diet, followed by macrophages, B, T, and NK cells (43).

In response to energy increase, adipocytes undergo hypertrophy, hyperplasia, and die, releasing in the extracellular space their cytoplasmic content including the lipid droplets, which cause the release of danger-associated molecular patterns such as free fatty acids, excess glucose, ATP, ceramides, cholesterol. All these activate macrophages expressing TLRs and NLRs, activate the inflammasome and initiate the AT inflammatory response, leading to the recruitment of monocytes, and increased polarization of macrophages to an inflammatory M1-like phenotype. Macrophages represent the primary source of TNF-α in the AT (50).

Neutrophils promote IR through the release of elastase (51), myeloperoxidase, and extracellular traps (ETs) (52). Aberrant production and reduced clearance of ETs can lead to accumulation of immunogenic self-antigens and promotion of autoimmune diseases (53).

NK cells significantly increase in number in the AT of mice fed with a high-fat diet. NK cells regulate the number and the function of AT macrophages through production of pro-inflammatory cytokines, mainly TNF-α, and thereby contribute to the development of IR. Depletion of NK cells using neutralizing antibodies has been shown to protect from IR (54).

Innate lymphoid cells have also been shown to promote IR, in particular ILC1s, which trigger M1 macrophage activation and inhibit ILC2 function through IFN-γ, thereby contributing to chronic inflammation and possibly perpetuating obesity-associated IR (55, 56).

In obese individuals, pro-inflammatory Th1 cells infiltrate the AT (57) and activate M1 macrophages (58), whereas in lean individuals Th2 cells, T regulatory, and iNKT cells are predominant in the VAT and promote secretion of IL-10 and other anti-inflammatory cytokines from M2 macrophages which maintain insulin sensitivity. The abdominal SAT has been reported to be dangerous as well in promoting inflammation (59).

**Figure 1:**
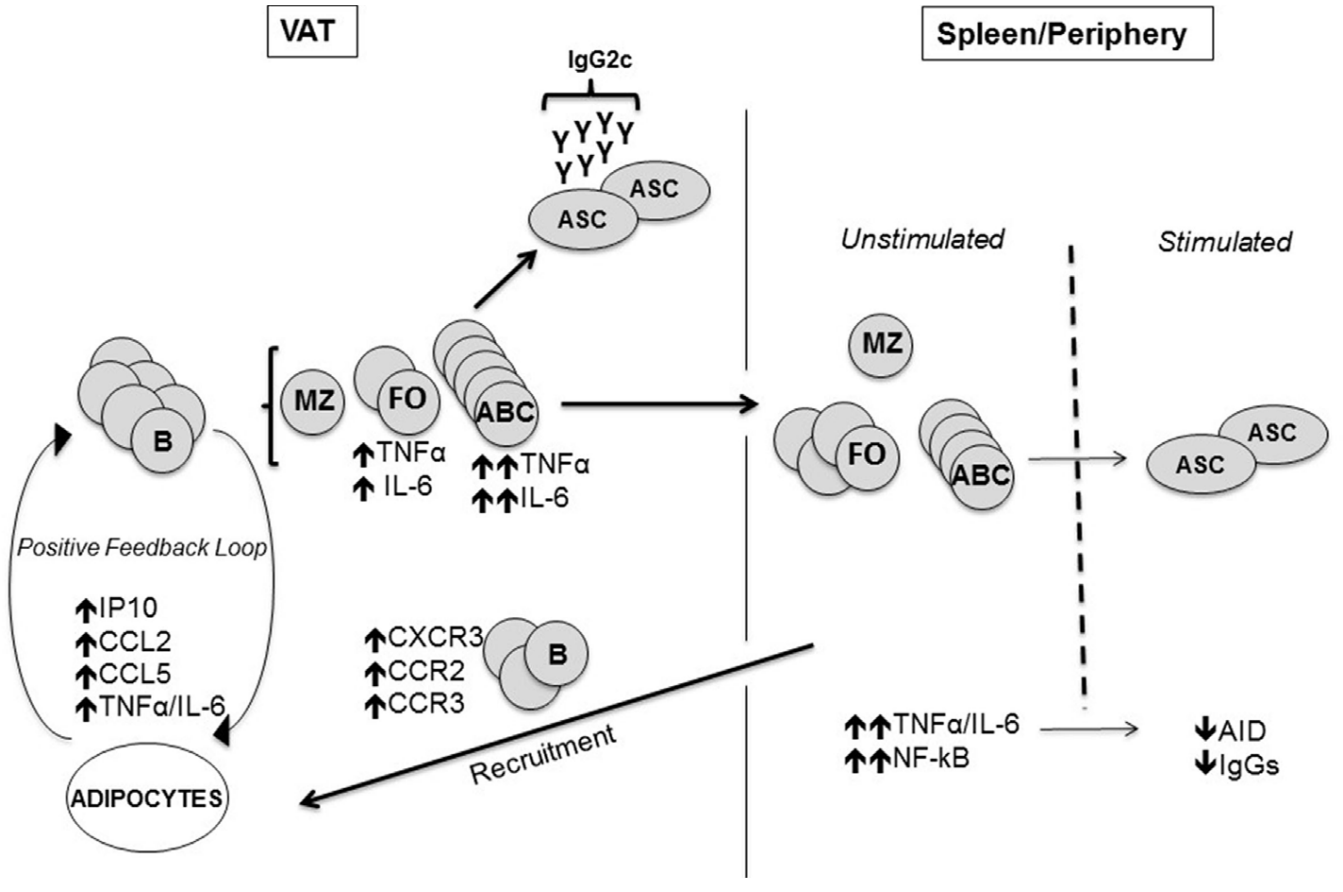
Mechanisms by which the visceral adipose tissue (VAT) impairs antibody responses. The adipocytes in the VAT secrete more pro-inflammatory chemokines which attract B cells *via* chemokine receptors, as well as pro-inflammatory cytokines. Age-associated B cells (ABCs) are preferentially induced and we hypothesize that these cells make pro-inflammatory cytokines and pathogenic antibodies. Marginal zone B cells are not affected. Immune cells traffic to the spleen and periphery where there are more ABC in aged and obese mice. Before antigen stimulation the cells secrete increased amounts of inflammatory cytokines (TNF-α/IL-6), making them refractory to further stimulation. Fewer antibody-secreting cells producing less Ig are made after antigen stimulation of “refractory” B cells.

Studies elucidating B cell function in obesity are limited, although B cells have recently emerged as crucial players in regulating inflammation in murine AT, by presenting antigens to T cells, secreting pro-inflammatory cytokines, and pathogenic antibodies (43). In mice, B2 cells accumulate in the AT before T cells, shortly after the initiation of a high-fat diet (60). We have recently shown that the adipocytes in murine VAT make several pro-inflammatory chemokines (CXCL10/CCL2/CCL5), which may recruit B2 cells as they express the corresponding receptors (CXCR3/CCR2/CCR3) (61). We believe that B2 cells infiltrating the VAT become more inflammatory and these cells would generate sub-optimal immune responses once they circulate back to the peripheral lymphoid tissues (Figure 1). B2 cells may also be recruited to the AT through leukotriene LTB4/LTB4R1 signaling (62).

B1 cells can also be found in the AT, although at lower percentages when compared with B2 cells, with B1a (but not B1b) being increased in the AT mice fed high-fat diet (43). These cells secrete IgM antibodies which have no effect on metabolic parameters in contrast with IgG, but they clear self-antigens and therefore have a regulatory role by limiting B2 cell activation and by promoting B cell tolerance. B10 producing B1 cells in the AT have been shown to have protective effects against diet-induced obesity and IR (63).

The early recruitment of B cells promotes T cell activation and pro-inflammatory cytokine production (43). B cells are activated in the expanding AT by pro-inflammatory stimuli and release cytokines or chemokines, thus contributing to local and systemic inflammation (64, 65). Antibodies secreted by B cells can also regulate lipid absorption from the gut and B^null^ mice show reduction in lipid absorption (66). This role of B cells in lipid adsorption could also shape mucosal immunity and change the gut microbiota (43, 67). Moreover, murine (44) and human B cells (39) support T cell inflammation.

The ongoing apoptosis in the AT, due to increases in fat mass, and consequent hypoxia, induces the release of “self” antigens, including cell-free DNA, and the release of class switched IgG antibodies which form immune complexes with “self” antigens, which in turn activate complement (C1q/C1qR/C3/C3a) and Fc receptors on immune cells, leading to enhanced local inflammation, remodeling of the AT, impairment of adipocyte function and of nutrient metabolism, and exacerbation of obesity-associated conditions. This represents a novel mechanism by which DNA released from cells dying in the AT may attract immune cells expressing TLRs, which may propagate the inflammatory response, as recently shown in mouse macrophages (68).

Obesity is also associated with altered composition of the gut microbiota, increased intestinal permeability, and translocation of gut bacterial products into the blood. These include lipopolysaccharide and unmethylated CpG DNA, which may exert effects systemically or locally in the AT (69, 70). Aberrant production and recognition of nucleic acid antigens has been suggested to promote activation of immune cells in metabolic tissues, leading to the secretion of pro-inflammatory cytokines (71).

## How the Adipose Tissue Affects Antibody Responses?

Obesity is associated with increased susceptibility to bacterial, viral, and fungal infections (72, 73) and obese individuals develop more post-surgical infections than lean individuals (74, 75). Moreover, overweight children have impaired antibody responses to tetanus toxin (76). Similarly, high-fat diet has been shown to increase mortality in mice infected with influenza (37). Contributing mechanisms seem to be defects in the generation and maintenance of memory CD8^+^ T cells (77), as well as impaired lung wound healing (78). The response to the influenza vaccine (39, 79) and to the Hepatitis B vaccine (80) are also compromised in individuals with obesity.

B cell function has been shown to be affected by leptin, the adipocyte-derived cytokine, member of the IL-6 superfamily, linking nutritional status with neuroendocrine, and immune functions, whose plasma levels correlate with the amount of body fat and BMI. The role of leptin in inflammation is supported by the studies in *ob/ob* mice which are leptin-deficient and have reduced secretion of Th1 cytokines (IL-2/IFN-γ/TNF-α/IL-18), and increased production of Th2 cytokines (IL-4/IL-10) (81–84). Leptin suppresses appetite in mice and humans (85, 86). High-serum levels of leptin contribute to the inflammatory state of the adipose tissue associated with obesity (87, 88). In individuals with obesity, leptin levels are associated with leptin resistance (86). Leptin activates human peripheral blood B cells from both young and elderly individuals to secrete pro-inflammatory cytokines (IL-6/TNF-α) and this occurs through activation of JAK2/STAT3 and p38MAPK/ERK1/2 signaling pathways (89, 90). In our recently published article, we stimulated B cells from lean individuals *in vitro* with leptin. We found pro-inflammatory signaling pathways upregulated (phospho-STAT3, crucial for TNF-α production) and anti-inflammatory pathways down-regulated (phospho-AMPK, crucial for antibody production), similar to what we observed in B cells from individuals with obesity (39).

We have recently discovered further mechanisms through which AT inflammation contributes to decreased B cell responses in old mice (61). We found AID in stimulated splenic B cells negatively correlated with epididymal fat size, showing for the first time a role of AT in the down-regulation of B cell function in aged mice. When we measured the percentages of the major peripheral B cell subsets [follicular (FO), age-associated B cells (ABCs), and marginal zone] in the spleens and epididymal VAT of young and old mice, we found reduced percentages of the FO subset in the spleen of old versus young mice and concomitant increased percentages of the pro-inflammatory ABC subset as previously shown (29, 91–93). Importantly, percentages of FO were reduced (and percentages of ABC were increased) even more in VAT versus spleen.

We have recently shown that the VAT promotes the differentiation of FO into ABCs (61). We demonstrated this by co-culturing in transwells for 72 h adipocytes and splenic B cells, in the absence of any additional mitogenic stimulus. Results showed increased percentages of ABC, which was similar to what we have observed in the VAT. To clarify if this was the result of increased expansion and survival of ABC versus FO B cells, death of FO B cells, loss of cell markers, or a combination of these, we sorted splenic FO and ABC from old mice and measured by qPCR the expression of several markers described to be differentially expressed in these two subsets by a transcriptome analysis performed previously by the Marrack group (92). We selected five markers among those most differentially expressed in FO versus ABC: Prdm1 (Blimp-1), FcεRγ1, Tbx21 (T-bet), Kifc3, Stx3. All these markers were found expressed at higher levels in ABC versus FO, as expected. Then, we cultured sorted splenic FO B cells from old mice in the presence of adipocyte-conditioned medium (ACM) and we found that the ACM induced significant increased expression of ABC markers when compared with complete medium, suggesting that FO B cells differentiated into ABC. In order to evaluate if the ACM contains factors which may be responsible for FO differentiation into ABC, as suggested by an article recently published by Cancro’s group (94), we measured by qPCR production of IL-21/IFN-γ by the adipocytes. Results showed that adipocytes express mRNA for both cytokines.

B cells have been shown to promote IR through activation of T cells and production of pro-inflammatory, pathogenic, autoimmune antibodies (43). We also found production of class switched IgG2c antibodies by B cells in the VAT, and these antibodies were detected by intracellular staining of VAT ABCs in the absence of stimulation, suggesting that ABC in the VAT are already highly pre-activated, and are refractory to further stimulation (61). IgG2c have been noted to be more autoimmune (95). Our results have shown for the first time that IgG2c antibodies are made in the VAT by ABCs, and the expression of MHC class I and class II on B cells has been reported to be crucial (43), suggesting that B cell-mediated antigen presentation to T cells is required for their pathogenic effects.

It has been shown that B cell depletion with an anti-CD20 antibody ameliorates metabolic disease, and transfer of IgG from high-fat diet mice rapidly induces IR (43). T cells can also be necessary for a pathogenic effect, as adoptive transfer of CD4^+^ T cells into high-fat diet RAG^null^ mice, lacking both B and T cells, was able to block weight gain and reverse IR for months, predominately through anti-inflammatory Th2 cells (96).

In conclusion, we have summarized emerging data on potential cellular and molecular mechanisms for the age-related increase in inflammation and how these lead to functional decline and decreased humoral responses in aged mice and humans. Overall, it appears that persistent inflammation is the driver of age-related diseases and that down regulation of inflammatory pathways may help to reduce onset and severity of age-related chronic diseases. Key challenges for the field will be to identify therapeutic strategies of intervention to lose weight will reduce body fat, systemic inflammation, and the pathogenic role of immune cells. Importantly, immune responses to fight infections will also be improved.

## Author Contributions

All authors were involved in writing the article and had final approval of the submitted and published versions.

## Conflict of Interest Statement

The research was conducted in the absence of any commercial or financial relationships that could be construed as a potential conflict of interest.

## References

[1] Boraschi D Del Giudice G Dutel C Ivanoff B Rappuoli R Grubeck-Loebenstein B. Ageing and immunity: addressing immune senescence to ensure healthy ageing. Vaccine (2010) 28(21):3627–31. 10.1016/j.vaccine.2010.03.035 20362616

[2] van Dijk-Hard I Soderstrom I Feld S Holmberg D Lundkvist I. Age-related impaired affinity maturation and differential D-JH gene usage in human VH6-expressing B lymphocytes from healthy individuals. Eur J Immunol (1997) 27(6):1381–6. 10.1002/eji.1830270613 9209488

[3] Gibson KL Wu YC Barnett Y Duggan O Vaughan R Kondeatis E B-cell diversity decreases in old age and is correlated with poor health status. Aging Cell (2009) 8(1):18–25. 10.1111/j.1474-9726.2008.00443.x 18986373 PMC2667647

[4] Khurana S Frasca D Blomberg B Golding H. AID activity in B cells strongly correlates with polyclonal antibody affinity maturation in-vivo following pandemic 2009-H1N1 vaccination in humans. PLoS Pathog (2012) 8(9):e1002920. 10.1371/journal.ppat.1002920 23028320 PMC3441753

[5] Goenka R Scholz JL Naradikian MS Cancro MP. Memory B cells form in aged mice despite impaired affinity maturation and germinal center kinetics. Exp Gerontol (2014) 54:109–15. 10.1016/j.exger.2013.12.013 24389058 PMC3989373

[6] Howard WA Gibson KL Dunn-Walters DK Antibody quality in old age. Rejuvenation Res (2006) 9(1):117–25. 10.1089/rej.2006.9.117 16608408

[7] Duggal NA Upton J Phillips AC Sapey E Lord JM. An age-related numerical and functional deficit in CD19(+) CD24(hi) CD38(hi) B cells is associated with an increase in systemic autoimmunity. Aging Cell (2013) 12(5):873–81. 10.1111/acel.12114 23755918 PMC3814412

[8] Naradikian MS Hao Y Cancro MP Age-associated B cells: key mediators of both protective and autoreactive humoral responses. Immunol Rev (2016) 269(1):118–29. 10.1111/imr.12380 26683149

[9] Pawelec G Barnett Y Forsey R Frasca D Globerson A McLeod J T cells and aging, January 2002 update. Front Biosci (2002) 7:d1056–183. 10.2741/A831 11991846

[10] Haynes L Eaton SM Burns EM Randall TD Swain SL. CD4 T cell memory derived from young naive cells functions well into old age, but memory generated from aged naive cells functions poorly. Proc Natl Acad Sci U S A (2003) 100(25):15053–8. 10.1073/pnas.2433717100 14657384 PMC299903

[11] Pawelec G Derhovanessian E. Role of CMV in immune senescence. Virus Res (2011) 157(2):175–9. 10.1016/j.virusres.2010.09.010 20869407

[12] Frasca D Van der Put E Riley RL Blomberg BB. Reduced Ig class switch in aged mice correlates with decreased E47 and activation-induced cytidine deaminase. J Immunol (2004) 172(4):2155–62. 10.4049/jimmunol.172.4.2155 14764681

[13] Frasca D Landin AM Lechner SC Ryan JG Schwartz R Riley RL Aging down-regulates the transcription factor E2A, activation-induced cytidine deaminase, and Ig class switch in human B cells. J Immunol (2008) 180(8):5283–90. 10.4049/jimmunol.180.8.5283 18390709

[14] van Duin D Mohanty S Thomas V Ginter S Montgomery RR Fikrig E Age-associated defect in human TLR-1/2 function. J Immunol (2007) 178(2):970–5. 10.4049/jimmunol.178.2.970 17202359

[15] Panda A Qian F Mohanty S van Duin D Newman FK Zhang L Age-associated decrease in TLR function in primary human dendritic cells predicts influenza vaccine response. J Immunol (2010) 184(5):2518–27. 10.4049/jimmunol.0901022 20100933 PMC3867271

[16] Frasca D Diaz A Romero M Landin AM Phillips M Lechner SC Intrinsic defects in B cell response to seasonal influenza vaccination in elderly humans. Vaccine (2010) 28(51):8077–84. 10.1016/j.vaccine.2010.10.023 20974306 PMC3223387

[17] Frasca D Diaz A Romero M Phillips M Mendez NV Landin AM Unique biomarkers for B-cell function predict the serum response to pandemic H1N1 influenza vaccine. Int Immunol (2012) 24(3):175–82. 10.1093/intimm/dxr123 22281510 PMC3621378

[18] Frasca D Diaz A Romero M Mendez NV Landin AM Blomberg BB. Effects of age on H1N1-specific serum IgG1 and IgG3 levels evaluated during the 2011-2012 influenza vaccine season. Immun Ageing (2013) 10(1):14. 10.1186/1742-4933-10-14 23607926 PMC3639840

[19] Frasca D Diaz A Romero M Landin AM Blomberg BB High TNF-alpha levels in resting B cells negatively correlate with their response. Exp Gerontol (2014) 54:116–22. 10.1016/j.exger.2014.01.004 24440385 PMC3989457

[20] Frasca D Diaz A Romero M Landin AM Blomberg BB Cytomegalovirus (CMV) seropositivity decreases B cell responses to the influenza vaccine. Vaccine (2015) 33(12):1433–9. 10.1016/j.vaccine.2015.01.071 25659271 PMC4352374

[21] Frasca D Diaz A Romero M Ferracci F Blomberg BB MicroRNAs miR-155 and miR-16 decrease AID and E47 in B cells from elderly individuals. J Immunol (2015) 195(5):2134–40. 10.4049/jimmunol.1500520 26223652 PMC4546853

[22] Sayegh CE Quong MW Agata Y Murre C. E-proteins directly regulate expression of activation-induced deaminase in mature B cells. Nat Immunol (2003) 4:586–93. 10.1038/ni923 12717431

[23] Franceschi C Valensin S Bonafe M Paolisso G Yashin AI Monti D The network and the remodeling theories of aging: historical background and new perspectives. Exp Gerontol (2000) 35(6–7):879–96. 10.1016/S0531-5565(00)00172-8 11053678

[24] Lindholm E Bakhtadze E Cilio C Agardh E Groop L Agardh CD. Association between LTA, TNF and AGER polymorphisms and late diabetic complications. PLoS One (2008) 3(6):e2546. 10.1371/journal.pone.0002546 18575614 PMC2429972

[25] Mundy GR. Osteoporosis and inflammation. Nutr Rev (2007) 65(12 Pt 2):S147–51. 10.1111/j.1753-4887.2007.tb00353.x 18240539

[26] Holmes C Cunningham C Zotova E Woolford J Dean C Kerr S Systemic inflammation and disease progression in Alzheimer disease. Neurology (2009) 73(10):768–74. 10.1212/WNL.0b013e3181b6bb95 19738171 PMC2848584

[27] Isaacs JD. Therapeutic agents for patients with rheumatoid arthritis and an inadequate response to tumour necrosis factor-alpha antagonists. Expert Opin Biol Ther (2009) 9(12):1463–75. 10.1517/14712590903379494 19916731

[28] Sarzi-Puttini P Atzeni F Doria A Iaccarino L Turiel M Tumor necrosis factor-alpha, biologic agents and cardiovascular risk. Lupus (2005) 14(9):780–4. 10.1191/0961203305lu2220oa 16218487

[29] Frasca D Romero M Diaz A Alter-Wolf S Ratliff M Landin AM A molecular mechanism for TNF-alpha-mediated downregulation of B cell responses. J Immunol (2012) 188(1):279–86. 10.4049/jimmunol.1003964 22116831 PMC3700394

[30] Hotamisligil GS Inflammation and metabolic disorders. Nature (2006) 444(7121):860–7. 10.1038/nature05485 17167474

[31] Shoelson SE Lee J Goldfine AB Inflammation and insulin resistance.J Clin Invest (2006) 116(7):1793–801. 10.1172/JCI29069 16823477 PMC1483173

[32] Johnson AM Olefsky JM The origins and drivers of insulin resistance. Cell (2013) 152(4):673–84. 10.1016/j.cell.2013.01.041 23415219

[33] Renehan AG Tyson M Egger M Heller RF Zwahlen M. Body-mass index and incidence of cancer: a systematic review and meta-analysis of prospective observational studies. Lancet (2008) 371(9612):569–78. 10.1016/S0140-6736(08)60269-X 18280327

[34] Setty AR Curhan G Choi HK. Obesity, waist circumference, weight change, and the risk of psoriasis in women: Nurses’ Health Study II. Arch Intern Med (2007) 167(15):1670–5. 10.1001/archinte.167.15.1670 17698691

[35] Casas R Sacanella E Estruch R. The immune protective effect of the mediterranean diet against chronic low-grade inflammatory diseases. Endocr Metab Immune Disord Drug Targets (2014) 14(4):245–54. 10.2174/1871530314666140922153350 25244229 PMC4443792

[36] Hass DJ Brensinger CM Lewis JD Lichtenstein GR The impact of increased body mass index on the clinical course of Crohn’s disease. Clin Gastroenterol Hepatol (2006) 4(4):482–8. 10.1016/j.cgh.2005.12.015 16616354

[37] Smith AG Sheridan PA Harp JB Beck MA. Diet-induced obese mice have increased mortality and altered immune responses when infected with influenza virus. J Nutr (2007) 137(5):1236–43. 17449587 10.1093/jn/137.5.1236

[38] Milner JJ Sheridan PA Karlsson EA Schultz-Cherry S Shi Q Beck MA. Diet-induced obese mice exhibit altered heterologous immunity during a secondary 2009 pandemic H1N1 infection. J Immunol (2013) 191(5):2474–85. 10.4049/jimmunol.1202429 23904168 PMC3756476

[39] Frasca D Ferracci F Diaz A Romero M Lechner S Blomberg BB. Obesity decreases B cell responses in young and elderly individuals. Obesity (Silver Spring) (2016) 24(3):615–25. 10.1002/oby.21383 26857091 PMC4769695

[40] Ibrahim MM. Subcutaneous and visceral adipose tissue: structural and functional differences. Obes Rev (2010) 11(1):11–8. 10.1111/j.1467-789X.2009.00623.x 19656312

[41] Yang YK Chen M Clements RH Abrams GA Aprahamian CJ Harmon CM. Human mesenteric adipose tissue plays unique role versus subcutaneous and omental fat in obesity related diabetes. Cell Physiol Biochem (2008) 22(5–6):531–8. 10.1159/000185527 19088435

[42] Wu D Ren Z Pae M Guo W Cui X Merrill AH Aging up-regulates expression of inflammatory mediators in mouse adipose tissue. J Immunol (2007) 179(7):4829–39. 10.4049/jimmunol.179.7.4829 17878382

[43] Winer DA Winer S Shen L Wadia PP Yantha J Paltser G B cells promote insulin resistance through modulation of T cells and production of pathogenic IgG antibodies. Nat Med (2011) 17(5):610–7. 10.1038/nm.2353 21499269 PMC3270885

[44] DeFuria J Belkina AC Jagannathan-Bogdan M Snyder-Cappione J Carr JD Nersesova YR B cells promote inflammation in obesity and type 2 diabetes through regulation of T-cell function and an inflammatory cytokine profile. Proc Natl Acad Sci U S A (2013) 110(13):5133–8. 10.1073/pnas.1215840110 23479618 PMC3612635

[45] van Harmelen V Skurk T Rohrig K Lee YM Halbleib M Aprath-Husmann I Effect of BMI and age on adipose tissue cellularity and differentiation capacity in women. Int J Obes Relat Metab Disord (2003) 27(8):889–95. 10.1038/sj.ijo.0802314 12861228

[46] Zamboni M Rossi AP Fantin F Zamboni G Chirumbolo S Zoico E Adipose tissue, diet and aging. Mech Ageing Dev (2014) 136–137:129–37. 10.1016/j.mad.2013.11.008 24321378

[47] Tchkonia T Morbeck DE Von Zglinicki T Van Deursen J Lustgarten J Scrable H Fat tissue, aging, and cellular senescence. Aging Cell (2010) 9(5):667–84. 10.1111/j.1474-9726.2010.00608.x 20701600 PMC2941545

[48] Lee M Martin H Firpo MA Demerath EW. Inverse association between adiposity and telomere length: the Fels Longitudinal Study. Am J Hum Biol (2011) 23(1):100–6. 10.1002/ajhb.21109 21080476 PMC3245638

[49] Grant RW Dixit VD. Adipose tissue as an immunological organ. Obesity (Silver Spring) (2015) 23(3):512–8. 10.1002/oby.21003 25612251 PMC4340740

[50] Weisberg SP McCann D Desai M Rosenbaum M Leibel RL Ferrante AW Jr. Obesity is associated with macrophage accumulation in adipose tissue. J Clin Invest (2003) 112(12):1796–808. 10.1172/JCI19246 14679176 PMC296995

[51] Talukdar S Oh DY Bandyopadhyay G Li D Xu J McNelis J Neutrophils mediate insulin resistance in mice fed a high-fat diet through secreted elastase. Nat Med (2012) 18(9):1407–12. 10.1038/nm.2885 22863787 PMC3491143

[52] Brinkmann V Reichard U Goosmann C Fauler B Uhlemann Y Weiss DS Neutrophil extracellular traps kill bacteria. Science (2004) 303(5663):1532–5. 10.1126/science.1092385 15001782

[53] Hakkim A Furnrohr BG Amann K Laube B Abed UA Brinkmann V Impairment of neutrophil extracellular trap degradation is associated with lupus nephritis. Proc Natl Acad Sci U S A (2010) 107(21):9813–8. 10.1073/pnas.0909927107 20439745 PMC2906830

[54] Lee BC Kim MS Pae M Yamamoto Y Eberle D Shimada T Adipose natural killer cells regulate adipose tissue macrophages to promote insulin resistance in obesity. Cell Metab (2016) 23(4):685–98. 10.1016/j.cmet.2016.03.002 27050305 PMC4833527

[55] Yang D Yang W Tian Z van Velkinburgh JC Song J Wu Y Innate lymphoid cells as novel regulators of obesity and its-associated metabolic dysfunction. Obes Rev (2016) 17(6):485–98. 10.1111/obr.12397 26948388

[56] Boulenouar S Michelet X Duquette D Alvarez D Hogan AE Dold C Adipose type one innate lymphoid cells regulate macrophage homeostasis through targeted cytotoxicity. Immunity (2017) 46(2):273–86. 10.1016/j.immuni.2017.01.008 28228283

[57] Nishimura S Manabe I Nagasaki M Eto K Yamashita H Ohsugi M CD8+ effector T cells contribute to macrophage recruitment and adipose tissue inflammation in obesity. Nat Med (2009) 15(8):914–20. 10.1038/nm.1964 19633658

[58] Lumeng CN Bodzin JL Saltiel AR. Obesity induces a phenotypic switch in adipose tissue macrophage polarization. J Clin Invest (2007) 117(1):175–84. 10.1172/JCI29881 17200717 PMC1716210

[59] McLaughlin T Deng A Yee G Lamendola C Reaven G Tsao PS Inflammation in subcutaneous adipose tissue: relationship to adipose cell size. Diabetologia (2010) 53(2):369–77. 10.1007/s00125-009-1496-3 19816674 PMC6290757

[60] Duffaut C Galitzky J Lafontan M Bouloumie A. Unexpected trafficking of immune cells within the adipose tissue during the onset of obesity. Biochem Biophys Res Commun (2009) 384(4):482–5. 10.1016/j.bbrc.2009.05.002 19422792

[61] Frasca D Diaz A Romero M Vazquez T Blomberg BB. Obesity induces pro-inflammatory B cells and impairs B cell function in old mice. Mech Ageing Dev (2017) 162:91–9. 10.1016/j.mad.2017.01.004 28111127 PMC5560850

[62] Ying W Wollam J Ofrecio JM Bandyopadhyay G El Ouarrat D Lee YS Adipose tissue B2 cells promote insulin resistance through leukotriene LTB4/LTB4R1 signaling. J Clin Invest (2017) 127(3):1019–30. 10.1172/JCI90350 28192375 PMC5330737

[63] Wu L Parekh VV Hsiao J Kitamura D Van Kaer L. Spleen supports a pool of innate-like B cells in white adipose tissue that protects against obesity-associated insulin resistance. Proc Natl Acad Sci U S A (2014) 111(43):E4638–47. 10.1073/pnas.1324052111 25313053 PMC4217427

[64] Nikolajczyk BS. B cells as under-appreciated mediators of non-auto-immune inflammatory disease. Cytokine (2010) 50(3):234–42. 10.1016/j.cyto.2010.02.022 20382544 PMC2917985

[65] Nikolajczyk BS Jagannathan-Bogdan M Shin H Gyurko R. State of the union between metabolism and the immune system in type 2 diabetes. Genes Immun (2011) 12(4):239–50. 10.1038/gene.2011.14 21390053 PMC6826342

[66] Shulzhenko N Morgun A Hsiao W Battle M Yao M Gavrilova O Crosstalk between B lymphocytes, microbiota and the intestinal epithelium governs immunity versus metabolism in the gut. Nat Med (2011) 17(12):1585–93. 10.1038/nm.2505 22101768 PMC3902046

[67] Winer DA Winer S Chng MH Shen L Engleman EG B Lymphocytes in obesity-related adipose tissue inflammation and insulin resistance. Cell Mol Life Sci (2014) 71(6):1033–43. 10.1007/s00018-013-1486-y 24127133 PMC3954849

[68] Nishimoto S Fukuda D Higashikuni Y Tanaka K Hirata Y Murata C Obesity-induced DNA released from adipocytes stimulates chronic adipose tissue inflammation and insulin resistance. Sci Adv (2016) 2(3):e1501332. 10.1126/sciadv.1501332 27051864 PMC4820373

[69] Henao-Mejia J Elinav E Jin C Hao L Mehal WZ Strowig T Inflammasome-mediated dysbiosis regulates progression of NAFLD and obesity. Nature (2012) 482(7384):179–85. 10.1038/nature10809 22297845 PMC3276682

[70] Winer DA Luck H Tsai S Winer S. The intestinal immune system in obesity and insulin resistance. Cell Metab (2016) 23(3):413–26. 10.1016/j.cmet.2016.01.003 26853748

[71] Revelo XS Ghazarian M Chng MH Luck H Kim JH Zeng K Nucleic acid-targeting pathways promote inflammation in obesity-related insulin resistance. Cell Rep (2016) 16(3):717–30. 10.1016/j.celrep.2016.06.024 27373163 PMC6354586

[72] Karlsson EA Beck MA. The burden of obesity on infectious disease. Exp Biol Med (Maywood) (2010) 235(12):1412–24. 10.1258/ebm.2010.010227 21127339

[73] O’Shea D Corrigan M Dunne MR Jackson R Woods C Gaoatswe G Changes in human dendritic cell number and function in severe obesity may contribute to increased susceptibility to viral infection. Int J Obes (Lond) (2013) 37(11):1510–3. 10.1038/ijo.2013.16 23439322

[74] Choban PS Flancbaum L The impact of obesity on surgical outcomes: a review. J Am Coll Surg (1997) 185(6):593–603. 10.1016/S1072-7515(97)00109-9 9404886

[75] Anaya DA Dellinger EP. The obese surgical patient: a susceptible host for infection. Surg Infect (Larchmt) (2006) 7(5):473–80. 10.1089/sur.2006.7.473 17083313

[76] Eliakim A Schwindt C Zaldivar F Casali P Cooper DM. Reduced tetanus antibody titers in overweight children. Autoimmunity (2006) 39(2):137–41. 10.1080/08916930600597326 16698670 PMC4623573

[77] Karlsson EA Sheridan PA Beck MA. Diet-induced obesity impairs the T cell memory response to influenza virus infection. J Immunol (2010) 184(6):3127–33. 10.4049/jimmunol.0903220 20173021

[78] O’Brien KB Vogel P Duan S Govorkova EA Webby RJ McCullers JA Impaired wound healing predisposes obese mice to severe influenza virus infection. J Infect Dis (2012) 205(2):252–61. 10.1093/infdis/jir729 22147799 PMC3244366

[79] Ovsyannikova IG White SJ Larrabee BR Grill DE Jacobson RM Poland GA. Leptin and leptin-related gene polymorphisms, obesity, and influenza A/H1N1 vaccine-induced immune responses in older individuals. Vaccine (2014) 32(7):881–7. 10.1016/j.vaccine.2013.12.009 24360890 PMC3922536

[80] Fan W Chen XF Shen C Guo ZR Dong C. Hepatitis B vaccine response in obesity: a meta-analysis. Vaccine (2016) 34(40):4835–41. 10.1016/j.vaccine.2016.08.027 27546877

[81] Loffreda S Yang SQ Lin HZ Karp CL Brengman ML Wang DJ Leptin regulates proinflammatory immune responses. FASEB J (1998) 12(1):57–65. 9438411

[82] Santos-Alvarez J Goberna R Sanchez-Margalet V. Human leptin stimulates proliferation and activation of human circulating monocytes. Cell Immunol (1999) 194(1):6–11. 10.1006/cimm.1999.1490 10357875

[83] Martin-Romero C Santos-Alvarez J Goberna R Sanchez-Margalet V. Human leptin enhances activation and proliferation of human circulating T lymphocytes. Cell Immunol (2000) 199(1):15–24. 10.1006/cimm.1999.1594 10675271

[84] Matarese G Moschos S Mantzoros CS. Leptin in immunology. J Immunol (2005) 174(6):3137–42. 10.4049/jimmunol.174.6.3137 15749839

[85] Halaas JL Gajiwala KS Maffei M Cohen SL Chait BT Rabinowitz D Weight-reducing effects of the plasma protein encoded by the obese gene. Science (1995) 269(5223):543–6. 10.1126/science.7624777 7624777

[86] Hukshorn CJ Saris WH. Leptin and energy expenditure. Curr Opin Clin Nutr Metab Care (2004) 7(6):629–33. 10.1097/00075197-200411000-00007 15534430

[87] La Cava A Matarese G The weight of leptin in immunity. Nat Rev Immunol (2004) 4(5):371–9. 10.1038/nri1350 15122202

[88] John BJ Irukulla S Abulafi AM Kumar D Mendall MA Systematic review: adipose tissue, obesity and gastrointestinal diseases. Aliment Pharmacol Ther (2006) 23(11):1511–23. 10.1111/j.1365-2036.2006.02915.x 16696799

[89] Agrawal S Gollapudi S Su H Gupta S Leptin activates human B cells to secrete TNF-alpha, IL-6, and IL-10 via JAK2/STAT3 and p38MAPK/ERK1/2 signaling pathway. J Clin Immunol (2011) 31(3):472–8. 10.1007/s10875-010-9507-1 21243519 PMC3132280

[90] Gupta S Agrawal S Gollapudi S. Increased activation and cytokine secretion in B cells stimulated with leptin in aged humans. Immun Ageing (2013) 10(1):3. 10.1186/1742-4933-10-3 23343052 PMC3557206

[91] Hao Y O’Neill P Naradikian MS Scholz JL Cancro MP. A B-cell subset uniquely responsive to innate stimuli accumulates in aged mice. Blood (2011) 118(5):1294–304. 10.1182/blood-2011-01-330530 21562046 PMC3152496

[92] Rubtsov AV Rubtsova K Fischer A Meehan RT Gillis JZ Kappler JW Toll-like receptor 7 (TLR7)-driven accumulation of a novel CD11c(+) B-cell population is important for the development of autoimmunity. Blood (2011) 118(5):1305–15. 10.1182/blood-2011-01-331462 21543762 PMC3152497

[93] Ratliff M Alter S Frasca D Blomberg BB Riley RL In senescence, age-associated B cells secrete TNFalpha and inhibit survival of B-cell precursors. Aging Cell (2013) 12(2):303–11. 10.1111/acel.12055 23410004 PMC3716274

[94] Naradikian MS Myles A Beiting DP Roberts KJ Dawson L Herati RS Cutting edge: IL-4, IL-21, and IFN-gamma interact to govern T-bet and CD11c expression in TLR-activated B cells. J Immunol (2016) 197(4):1023–8. 10.4049/jimmunol.1600522 27430719 PMC4975960

[95] Peng SL Szabo SJ Glimcher LH. T-bet regulates IgG class switching and pathogenic autoantibody production. Proc Natl Acad Sci U S A (2002) 99(8):5545–50. 10.1073/pnas.082114899 11960012 PMC122806

[96] Winer S Chan Y Paltser G Truong D Tsui H Bahrami J Normalization of obesity-associated insulin resistance through immunotherapy. Nat Med (2009) 15(8):921–9. 10.1038/nm.2001 19633657 PMC3063199

